# Environmental Impact of Food Preparations Enriched with Phenolic Extracts from Olive Oil Mill Waste

**DOI:** 10.3390/foods10050980

**Published:** 2021-04-29

**Authors:** Alessia Pampuri, Andrea Casson, Cristina Alamprese, Carla Daniela Di Mattia, Amalia Piscopo, Graziana Difonzo, Paola Conte, Maria Paciulli, Alessio Tugnolo, Roberto Beghi, Ernestina Casiraghi, Riccardo Guidetti, Valentina Giovenzana

**Affiliations:** 1 Department of Agricultural and Environmental Sciences (DiSAA), Università degli Studi di Milano, 20133 Milan, Italy; alessia.pampuri@unimi.it (A.P.); alessio.tugnolo@unimi.it (A.T.); roberto.beghi@unimi.it (R.B.); riccardo.guidetti@unimi.it (R.G.); valentina.giovenzana@unimi.it (V.G.); 2 Department of Food, Environmental, and Nutritional Sciences (DeFENS), Università degli Studi di Milano, 20133 Milan, Italy; cristina.alamprese@unimi.it (C.A.); ernestina.casiraghi@unimi.it (E.C.); 3 Faculty of Bioscience and Technology for Agriculture, Food and Environment, University of Teramo, 64100 Teramo, Italy; cdimattia@unite.it; 4 Department of Agraria, University Mediterranea of Reggio Calabria, 89124 Reggio Calabria, Italy; amalia.piscopo@unirc.it; 5 Department of Soil, Plant and Food Sciences, Food Science and Technology Unit, University of Bari Aldo Moro, 70126 Bari, Italy; graziana.difonzo@uniba.it; 6 Department of Agricultural Sciences, Università degli Studi di Sassari, 07100 Sassari, Italy; pconte@uniss.it; 7 Department of Food and Drug, University of Parma, 43124 Parma, Italy; maria.paciulli@unipr.it

**Keywords:** life cycle assessment, biocompounds, shelf life, environmental sustainability, biscuits, gluten-free breadsticks, salad dressing, vegan mayonnaise, circular economy, waste recovery

## Abstract

Reducing food waste as well as converting waste products into second-life products are global challenges to promote the circular economy business model. In this context, the aim of this study is to quantify the environmental impact of lab-scale food preparations enriched with phenolic extracts from olive oil mill waste, i.e., wastewater and olive leaves. Technological (oxidation induction time) and nutritional (total phenols content) parameters were considered to assess the environmental performance based on benefits deriving by adding the extracts in vegan mayonnaise, salad dressing, biscuits, and gluten-free breadsticks. Phenolic extraction, encapsulation, and addiction to the four food preparations were analyzed, and the input and output processes were identified in order to apply the life cycle assessment to quantify the potential environmental impact of the system analyzed. Extraction and encapsulation processes characterized by low production yields, energy-intensive and complex operations, and the partial use of chemical reagents have a non-negligible environmental impact contribution on the food preparation, ranging from 0.71% to 73.51%. Considering technological and nutritional aspects, the extraction/encapsulation process contributions tend to cancel out. Impacts could be reduced approaching to a scale-up process.

## 1. Introduction

According to the International Olive Council, the global production of olive oil has been constantly increasing from 1.8 million tons per year in the 1990s, and the production currently amounts to more than 3 million tons per year. Olive oil production represents a significant sector all over the world and in the European Union economy. EU countries contributed almost 70% of all olive oil produced in the world in the 2018–2019 harvest year campaign, and the resultant revenue was about five billion euro [1].

Italy is the second largest producer of olive oil after Spain. Most of the production (about 80%) is concentrated in Apulia, Sicily, and Calabria. On the other hand, in the northernmost regions, the climatic conditions have allowed the cultivation of olives since ancient times thanks to microclimates; however, the cultivation is much less widespread than in southern Italy [2].

Olive drupes and olive oil are potential sources of several bioactive compounds as phenolic compounds, tocopherols, and other antioxidants. During oil extraction, many of these secondary metabolites can be destroyed or degraded or transferred in olive oil mill waste. At the end of the extraction process, olive oil contains 1–2% of the total phenol content (TPC) of drupes, so the residual antioxidant compounds would be lost in olive mill wastewater and pomace [3].

In particular, the traditional press extraction method as well as the continuous three-phase decanter process, which is most widely used for the production of olive oil, generate three principal products: olive oil (20%) and two streams of waste, a wet solid waste (30%) called ‘‘crude olive cake’’ and an aqueous waste called ‘‘olive mill wastewater’’ (50%). The solid waste (crude olive cake) is the residue that remains after the first pressing of the olives and is a mixture of pomace, stones, leaves, and dust [4].

Despite the economic and nutritional importance of this food product in many countries, the olive oil industry causes diverse environmental impacts in terms of resource depletion, land degradation, air emissions, and waste generation. These impacts may vary as a result of the practices and techniques employed in olive cultivation and oil production, and they can also vary from one country to another and also from one region to another within the same geographical area [5].

Many olive mill waste products could be exploited as by-products to be used as fuels, fertilizers, or other intermediate products for the food, nutraceutical, cosmetic, and pharmaceutical industries. Olive pomace and olive oil mill wastewater could be considered a low-cost and renewable source of high-added value compounds [6].

These by-products are still undervalued even if they have a good potential as sources of bioactive components [7]: the phenolic compounds deriving from olive possess antimicrobial, anti-inflammatory, and chemopreventive properties [8,9]. Olive polyphenols have been proven to exert important technological functionality [10] such as a water/oil-holding capacity and emulsifying activity and can represent a useful ingredient that can help in the production and stabilization of complex food products such as emulsions [11].

However, the quantity and the specific characteristics of these by-products depends on climatic conditions and production practices. For these reasons nowadays, many studies are focusing on the management of by-products of the olive oil industry to try to further enhance this supply chain.

From a circular economy perspective, it is possible to assess, and in the future reduce, the environmental impact of these processes and waste products using a life cycle assessment analysis (LCA) [12]. There are several studies in the literature, mostly conducted in Mediterranean countries, in which the LCA of producing olive oil via different methods have been performed [5,13,14,15,16,17,18,19,20,21,22,23].

The aim of this study is to quantify the environmental impact of the extraction of bioactive phenolic compounds from olive oil mill wastewater and olive leaves. To evaluate the impact of phenols’ extraction and encapsulation processes within the entire supply chain, lab-scale food preparations were analyzed. Moreover, technological and nutritional parameters (oxidation induction time and total phenols content) were used to assess the environmental performance of production process based on benefits deriving from adding the phenolic extract in four food preparations: vegan mayonnaise, salad dressing, biscuits, and gluten-free breadsticks.

## 2. Materials and Methods

The phenolic extracts (PE) were obtained following two extraction methods, olive oil mill wastewater (OMWW) and from olive leaves (OL), to obtain olive leaves extract (OLE). PE were added to different food preparations, as free extracts of OMWW (OMWW PE) in vegan mayonnaise (mayo) and gluten-free breadsticks (GFB), as free extracts of OL (OLE) in gluten-free breadsticks, and as encapsulated OLE (eOLE) in salad dressing and biscuits.

Life cycle assessment is a standardized method aimed at evaluating the environmental impacts studying the whole cycle of a product or a service. It considers all the inputs and outputs from raw materials extraction until end-of-life scenarios of the product or service analyzed. The following LCA study was developed in compliance with the international standards of series ISO 14040 and ISO 14044 [12]. According to ISO standards [12], the analysis was articulated in four stages: goal and scope definition, life cycle inventory (LCI), life cycle impact assessment (LCIA), and interpretation of the results (proposed in the Section 4).

### 2.1. Goal and Scope Definition

The goal of this study is to quantify the potential environmental impact of the use of phenolic extracts (PE) from olive mill wastewater (OMWW) and from olive leaves (OL) in particular case studies of food processes to improve nutritional and technological parameters.

The functional unit (FU) defines the reference unit of the system under analysis (ISO 14040 and 14044) [12]. To evaluate the environmental impact of the polyphenol’s extraction processes and encapsulating process, 1 g of TPC was defined as FU.

Differently, a commercial unit was used to evaluate the weight of the PE as ingredients in four food formulations: (i) 350 g for vegan mayonnaise, (ii) 135 mL for salad dressing, (iii) 160 g for biscuits, and (iv) 300 g for gluten free breadsticks.

Moreover, the commercial FU for each product was normalized considering technological and nutritional parameters.

**Figure 1:**
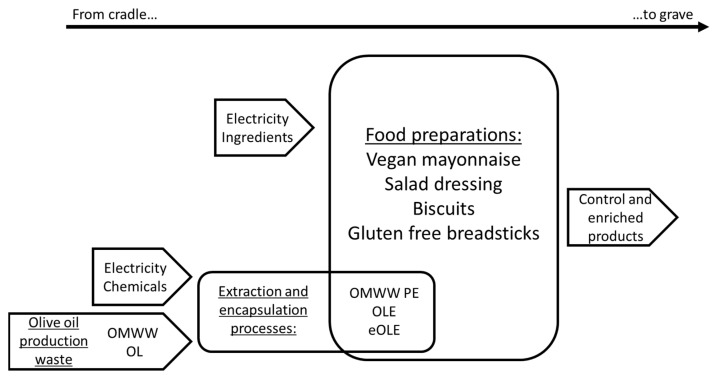
Simplified system boundaries.

The system under study follows an approach called “from cradle to grave” where all the factors were considered from the olive oil extraction process to the formulation of the four food preparations. In detail, as reported in Figure 1 (detailed system boundaries are reported in supplementary data Figure S1), every input (extraction of raw materials, energy and water consumption, chemicals) and output (hazardous waste and food production waste) were considered. Regarding the food preparations, the consumption and the packaging were neglected.

#### 2.1.1. Normalization Factors

Since the PE addiction implies advantages in term of technological and nutritional aspects, the environmental impact calculated considering commercial FU was normalized based on Total Phenolic Content (TPC) and oxidative stability. In order to quantify (i) the TPC, Singleton and Rossi’s method [13] was performed and (ii) the oxidative stability method used by Paciulli et al. [14] was carried out. Gallic acid equivalent expressed as TPC (mg/g) and oxidative stability expressed in oxidation induction days represent the units describing nutritional and technological parameters respectively.

In this work different combinations of extracts (OMWW PE, OLE, and eOLE) and food preparations were considered and only the best performing enriched food preparations were studied (higher values of induction days and mg of TPC/g of product) and reported in Table 1. To evaluate the technological and nutritional performance of enriched food preparations, a conventional production process (control) was also analyzed.

#### 2.1.2. Allocation Criteria

Considering the olive oil mill process and according to Parascanu et al. [20], olive oil has a much higher economic value compared to the other by-products, not only representing the higher output in terms of product amount (mass allocation in Table 2). To better evaluate the environmental impact of every olive oil mill output, an economic allocation was used. In particular, according to Tsagaraki et al. [4], in addition to the conventional subdivision of the by-products, it was possible to identify in a more specific way the average composition of the different outputs: (i) pomace, (ii) wastewater, (iii) stone, and (iv) leaves and dust (Table 2).

Details about the transition from the mass allocation to the economic one was reported in Table 2. Overall, OMWW and OL values represent a cost for the milling process instead of a revenue, not having economic allocation (waste products). In this work, in order to calculate the relative environmental impact, a minimum economic value attribution was chosen also for these products (by-products). Considering the four food preparations, a mass allocation was used.

### 2.2. Life Cycle Inventory (LCI)

Regarding the olive oil milling process, secondary data from the WFLD (World Food LCA Database) were used and modeled in to obtain the five different outputs as argued in the allocation criteria paragraph. OMWW and OL were used as inputs for the following extraction processes. The inventory of OMWW PE and OLE extraction phases, encapsulation phase (eOLE), and the related applications on food preparations, on the contrary, were performed separately and reported below using primary data.

#### 2.2.1. OMWW PE Extraction Phase LCI

Following the method used by Romeo et al. [24], 2 L of olive mill wastewater were acidified with 1 mL of HCl to obtain a pH 2 mixture. The mixture passed three cycles which required 2 L of hexane and 3 min of centrifuge (0.5 kW) for each cycle. Then, 0.625 L of ethyl acetate were added, and using (i) the centrifuge (3000 rpm 0.8 kW, 18 min) and (ii) the evaporator for 2 h, 3 g of dry residue were obtained. Then, 100 mL of water were added to the dry residue to obtain a solution, which was filtered to obtain 103 mL of OMWW PE. Hexane and ethyl acetate were recovered after use for 75%, and the remaining 25% were treated as hazardous waste. Input and output data related to this analysis and allocated to the FU (equivalent to 34.33 mL) are reported in Table 3.

#### 2.2.2. OLE Extraction Phase LCI

According to Difonzo et al. [25], 200 g of olive leaves were washed using 1 L of water, dried firstly with paper (2–3 pieces) and then using an oven (0.53 kW, 8 min, 120 °C). Then, 100 g of hot air-dried leaves (3–4% water content) were milled for 30 s using a mill (0.175 kW). The powder obtained passed three cycles, which required (i) 2L of water, (ii) the use of ultrasound (0.2 kW, 30 min), and (iii) filtering to obtain 6 L of filtered aqueous extract. Using a freeze dryer (1.4 kW, 24 h), 10 g of OLE were obtained. Then, 100 g of exhausted leaves were treated as biowaste. Input and output data related to this analysis and allocated to the FU (equivalent to 6.67 g) are reported in Table 4.

#### 2.2.3. eOLE Encapsulation Phase LCI

According to Flamminii et al. [26], 0.4 g of OLE, 0.4 g of pectin, 1.64 g of calcium citrate, 0.4 g of alginate, and 17.16 mL of water were mixed to 98 g of sunflower oil and 2 g of Span 80 (emulsifier) and agitated using a stirring plate (0.4 kW, 15 min). Then, 20 g of sunflower oil and 0.5 g of glacial acetic acid were added and agitated using a stirring plate (0.4 kW, 30 min). Then, 3 g of OLE, 145.42 mL of water, 0.83 g of calcium chloride, and 0.75 g of Tween20 (surfactant) were added to the mixture and agitated again using a stirring plate (0.4 kW, 30 min). To obtain the beads (92% water content and 8% dried matter), a centrifuge was used for 5 min (0.800 kW). Then, 30 mL of ethanol solution (21 mL ethanol, 8.94 mL water, and 0.6 g OLE) was added to obtain 20 g of cleaned beads. Lastly, the beads were lyophilized using a freeze drier for 24 h (1.4 kW) to obtain 1.60 g of eOLE. Then, 300.5 g of exhausted oil were treated as waste oil. Input and output data related to this analysis and allocated to the FU (1 g of TPC equivalent to 5 g of eOLE) are reported in Table 5.

#### 2.2.4. Vegan Mayonnaise LCI

For the traditional formulation (Mayo) used as control, 150 g of soy milk were mixed with 1 g of lemon juice and 199 g of sunflower oil using a blender (1 kW) for 4 min. The final product obtained weighed 350 g. Differently, for the product enriched with polyphenols (Mayo + OMWW PE), 100 g of soy milk were mixed with 1 g of lemon juice, 199 g of sunflower oil, and 50 g of OMWW PE using a blender (1 kW) for 4 min. In this case, the final product obtained weighted 350 g. Input and output data related to this preparation are reported in Table 6.

#### 2.2.5. Salad Dressing LCI

According to Jolayemi et al. [27], for the traditional formulation of salad dressing (salad dressing control), 99.55 mL of water were homogenized with 0.80 g of xanthan gum, 0.50 g of citric acid, 0.40 g of salt, and 35.75 mL of corn oil using a blender (0.75 kW) for 1 min to obtain 135 mL of salad dressing. Differently, for the product enriched with polyphenols (salad dressing + eOLE), 99.25 mL of water was mixed with 0.80 g of xanthan gum, 0.5 g of citric acid, and 0.4 g of salt. Then, 0.3 g of eOLE were added to the aqueous phase; finally, 33.75 mL of corn oil were added to obtain the salad dressing composed of 25% oil and 75% aqueous phase. Two blendings were carried out using a blender (0.75 kW) for 30 s each to obtain 135 mL of salad dressing. Input and output data related to this preparation are reported in Table 7.

#### 2.2.6. Biscuits LCI

According to Paciulli et al. [28], for the biscuits, 17 g of water, 1 g of salt, and 1 g baking powder were mixed with 100 g of soft wheat flour using a blender for 15 min (0.3 kW). Then, 30 g of butter and 50 g of icing sugar were blended for 3 min and then added to the dough and blended for another 6 min. The final dough (200 g), after a chilling phase of 10 min in a refrigerator, was formed manually to obtain 12 biscuits. After the cooking phase (180 °C; 20 min; 0.530 kW), 12 biscuits (13.5 g each) were obtained. For the enriched formulation (Biscuits + PE), 99.45 g of soft wheat flour and 0.55 g of eOLE were blended for 15 min (kW); the blended mixture was added to the solution of 17 g of water, 1 g of salt, and 1 g of baking powder. The other steps that follow are the same as for the traditional formulation. Input and output data related to this preparation are reported in Table 8.

#### 2.2.7. Gluten-Free Breadsticks LCI

To obtain the experimental gluten-free breadsticks, 500 g of rice flour, 500 g of corn starch, 550 mL of warm water (26 °C), 100 g of sunflower oil, 15 g of guar gum, 15 g of psyllium fiber, 30 g of sugar, 18 g of salt, and 40 g of compressed yeast were used as the basic formulation. For the preparation of the control samples, the dry ingredients were pre-blended (0.3 kW) for 2 min to ensure a proper homogenization and then mixed with sugar, salt, and yeast—previously dissolved in aliquots of water—and 100 g of sunflower oil for 13 min by using a professional mixer (0.3 kW). The resulting dough, after a leavening phase of 30 min (33 °C), was divided in 62 pieces of 28 g each and subjected to a second leavening (30 min; 33 °C). Then, the breadstick samples were baked (0.53 kW; 180 °C) for 13 min, rested for 30 min, and baked again for 22 min. Differently from the control breadsticks, the enriched ones were formulated using 1 g of PE from OMWW or OL (GFB + OMWW PE, GFB + OLE). Input and output data related to this preparation are reported in Table 9.

### 2.3. Life Cycle Impact Assessment (LCIA)

Life cycle impact assessment (LCIA) translates emissions and resource extractions into a limited number of environmental impact scores by means of characterization factors. These factors convert the data from LCI to the common unit of category indicator. According to Goedkoop et al. [29], the Recipe 2016 Midpoint (H) v1.04 method was used to assess the potential environmental impact. The LCIA data results were proposed using the following impact categories: Global warming (GW), Stratospheric ozone depletion (OD), Ionizing radiation (IR), Ozone formation, human health (OF-HH), Fine particulate matter formation (PM), Ozone formation, terrestrial ecosystems (OF-TE), Terrestrial acidification (TA), Freshwater eutrophication (FE), Marine eutrophication (ME), Terrestrial ecotoxicity (TE), Freshwater ecotoxicity (FRE), Marine ecotoxicity (MECO), Human carcinogenic toxicity (HCT), Human non-carcinogenic toxicity (HNCT), Land use (LU), Mineral resource scarcity (MRS), Fossil resource scarcity (FRS), and Water consumption (WC). Ecoinvent 3.6 (allocation, cut-off by classification) and World Food LCA Database 3.5 were used as databases for the inventory phase, SimaPro version 9.1.1. (PRè Sustainability, Amersfoort, The Netherlands) was used to assess the environmental impacts of PE extractions, PE encapsulation, and food preparations.

## 3. Results

The PE extractions, PE encapsulation process, and their use in four food formulation will be analyzed separately in the following sections.

### 3.1. PE Extraction and OLE Encapsulation Impact Assessment

**Figure 2:**
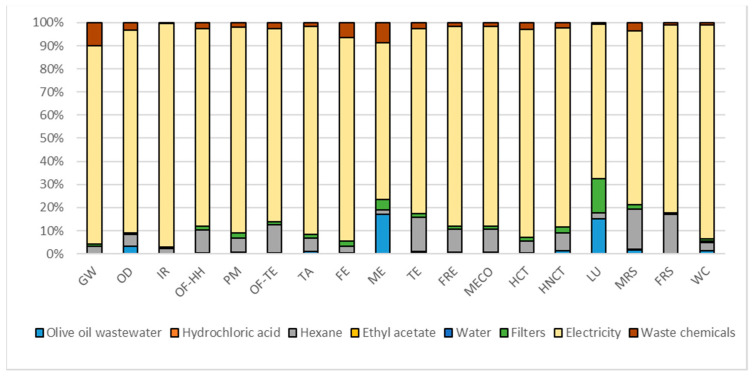
Hotspot deriving from contribution analysis related to OMWW PE. Global warming (GW), Stratospheric ozone depletion (OD), Ionizing radiation (IR), Ozone formation, human health (OF-HH), Fine particulate matter formation (PM), Ozone formation, terrestrial ecosystems (OF-TE), Terrestrial acidification (TA), Freshwater eutrophication (FE), Marine eutrophication (ME), Terrestrial ecotoxicity (TE), Freshwater ecotoxicity (FRE), Marine ecotoxicity (MECO), Human carcinogenic toxicity (HCT), Human non-carcinogenic toxicity (HNCT), Land use (LU), Mineral resource scarcity (MRS), Fossil resource scarcity (FRS), and Water consumption (WC).

**Figure 3:**
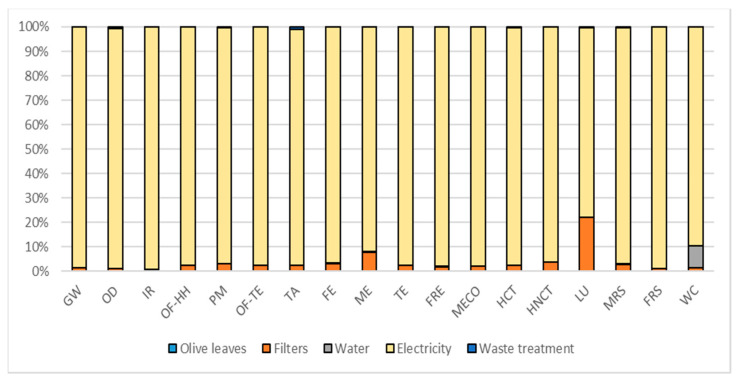
Hotspot deriving from contribution analysis related to OLE. Global warming (GW), Stratospheric ozone depletion (OD), Ionizing radiation (IR), Ozone formation, human health (OF-HH), Fine particulate matter formation (PM), Ozone formation, terrestrial ecosystems (OF-TE), Terrestrial acidification (TA), Freshwater eutrophication (FE), Marine eutrophication (ME), Terrestrial ecotoxicity (TE), Freshwater ecotoxicity (FRE), Marine ecotoxicity (MECO), Human carcinogenic toxicity (HCT), Human non-carcinogenic toxicity (HNCT), Land use (LU), Mineral resource scarcity (MRS), Fossil resource scarcity (FRS), and Water consumption (WC).

**Figure 4:**
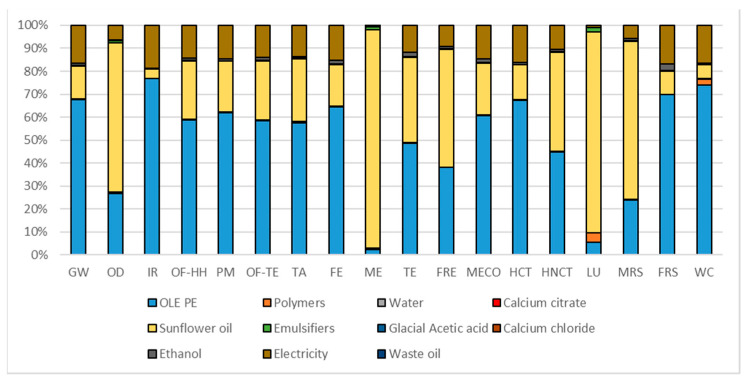
Hotspot deriving from contribution analysis related to eOLE. Global warming (GW), Stratospheric ozone depletion (OD), Ionizing radiation (IR), Ozone formation, human health (OF-HH), Fine particulate matter formation (PM), Ozone formation, terrestrial ecosystems (OF-TE), Terrestrial acidification (TA), Freshwater eutrophication (FE), Marine eutrophication (ME), Terrestrial ecotoxicity (TE), Freshwater ecotoxicity (FRE), Marine ecotoxicity (MECO), Human carcinogenic toxicity (HCT), Human non-carcinogenic toxicity (HNCT), Land use (LU), Mineral resource scarcity (MRS), Fossil resource scarcity (FRS), and Water consumption (WC).

Table 10 represents the overall potential environmental impacts while Figure 2, Figure 3 and Figure 4 report the contribution analysis related to the different PE extractions and encapsulation. According to the FU used for the extraction and encapsulation processes, OMWW PE represents the least environmental impactful extraction technique (6.69 times less). The encapsulation of OLE, to obtain eOLE, represents a 10 times more impactful process than the environmental impact of the extraction of 1 g TPC contained in OLE.

To highlight hotspots, details regarding the contribution analysis of the different factors influencing extraction and encapsulation processes were reported.

#### 3.1.1. Contribution Analysis of OMWW PE Impact Assessment

Factors influencing the OMWW PE extraction were reported in Figure 2. The factor electricity reached the highest values for all the impact categories from the lowest value reached in LU (67%) to the highest one reached in IR impact category (97%). This large contribution depends on the energy demand from the equipment necessary for the extraction of phenols from olive oil wastewater; the major contribution comes from the evaporator, which absorbs 86% of the overall energy consumption. Despite the tiny quantity used and allocated to this extraction process, the contribution of hexane covers an average weight among impact categories equal to 8% and thus represents the second contribution factor. The waste chemicals factor, which refers to the waste management of chemicals, represents the third contribution of this extraction technique, contributing with an average weight among impact categories of about 3%.

As for the OMWW PE, also for the OLE process (Figure 3), the electricity contributes mainly to all the impact categories with an average contribution of about 97%. This high level of contribution is directly linked to the energy consumption of the freeze dryer (97% of the total energy consumption). The second contribution factor is identified in the paper filters used, even if this factor has a contribution of about 4% among impact categories

Differently from the two extraction processes previously analyzed, Figure 4 shows a fragmented contribution analysis of the eOLE reporting different contribution factors. The highest contribution of the whole process comes from the OLE production process, which quantified the related weight of this factor equal to 50% among impact categories. The second contribution factor is the sunflower oil with an average weight among impact categories equal to 35%. This factor shows higher contribution in those impact categories related to the cultivation phases (ME 97%; LU 85%). The factor electricity contributes less compared to the other processes analyzed, but it anyway covers an average contribution of about 12%. In this case, the stirring plate requires 56% of the total energy consumption due to the time of usage, while the freeze dryer requires only 37% of the total energy consumption.

#### 3.1.2. Vegan Mayonnaises Impact Assessment

**Figure 5:**
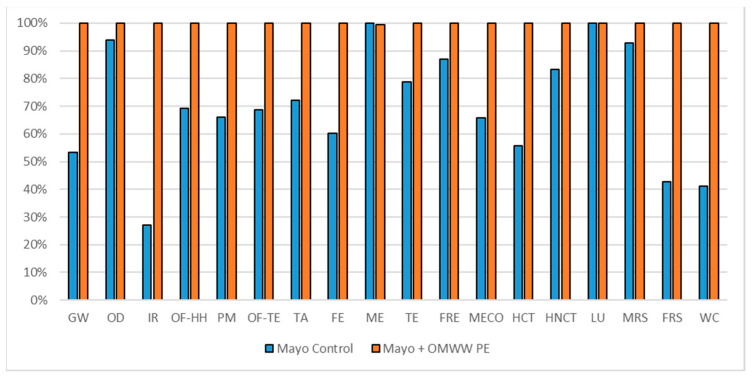
Environmental impact comparison of a vegan mayonnaise (350 g) vs. PE enriched product. Global warming (GW), Stratospheric ozone depletion (OD), Ionizing radiation (IR), Ozone formation, human health (OF-HH), Fine particulate matter formation (PM), Ozone formation, terrestrial ecosystems (OF-TE), Terrestrial acidification (TA), Freshwater eutrophication (FE), Marine eutrophication (ME), Terrestrial ecotoxicity (TE), Freshwater ecotoxicity (FRE), Marine ecotoxicity (MECO), Human carcinogenic toxicity (HCT), Human non-carcinogenic toxicity (HNCT), Land use (LU), Mineral resource scarcity (MRS), Fossil resource scarcity (FRS), and Water consumption (WC).

**Figure 6:**
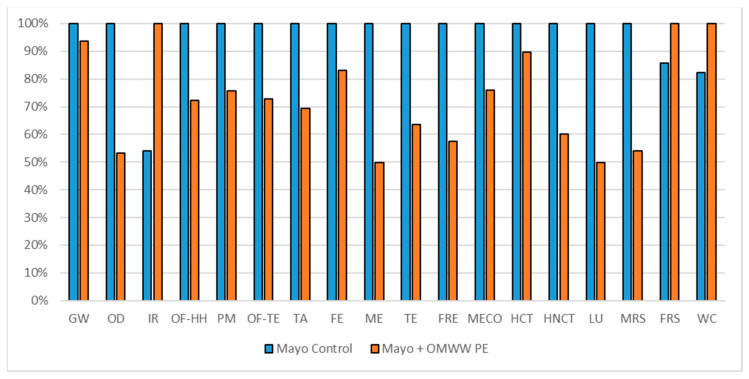
Environmental impact comparison of a vegan mayonnaise (350 g) vs. PE enriched product normalized to oxidation induction time. Global warming (GW), Stratospheric ozone depletion (OD), Ionizing radiation (IR), Ozone formation, human health (OF-HH), Fine particulate matter formation (PM), Ozone formation, terrestrial ecosystems (OF-TE), Terrestrial acidification (TA), Freshwater eutrophication (FE), Marine eutrophication (ME), Terrestrial ecotoxicity (TE), Freshwater ecotoxicity (FRE), Marine ecotoxicity (MECO), Human carcinogenic toxicity (HCT), Human non-carcinogenic toxicity (HNCT), Land use (LU), Mineral resource scarcity (MRS), Fossil resource scarcity (FRS), and Water consumption (WC).

The environmental impact comparison related to the formulation of 350 g of vegan mayonnaise and 350 g of enriched vegan mayonnaise is reported in Figure 5, while the comparison of environmental impact of the two formulations normalized to the shelf-life parameters is reported in Figure 6. The environmental impact results from these two analyses and TPC normalization factor was reported also in Table S1.

According to the comparison of the commercial unit (350 g of product for each formulation), the enriched product reports higher environmental impacts in most of the impact categories. As reported in Figure 2, the high demand of electricity required for the OMWW PE process can be identified in the differences between the traditional mayonnaise and the enriched one. The average percentage of responsibility related to the OMWW PE in the enriched formulation weights of about 30% (as reported in Table 11). The gap between the two products goes from a lower value equal to 0% in ME and LU (related mainly to the field activities of sunflower oil) to the higher value reached in IR, which counts 73% less in traditional formulation. Considering only the commercial unit, the traditional mayonnaise shows better environmental impact with respect to the enriched one (30% more convenient).

Different considerations must be done according to the estimated shelf-life values quantified in Table 1. The traditional mayonnaise, which claims the worst technological performances (half induction period) with respect to the enriched one, reports different results in Figure 6.

The induction days parameter was used to compare the potential environmental impact of the two preparations. The technological characteristics of the enriched mayonnaise (1 induction day) counted double values with respect to the traditional mayonnaise (0.5 induction days). Considering the worst-case scenario, the induction day cannot be identified as a representative parameter for a normalization of the environmental impact respect to the oxidation induction time. Despite this, potential food waste has been identified as a parameter for the technological performance of the two food preparations. Considering the potential shelf life, the enriched mayonnaise, which claims a double induction period with respect to the traditional one, reports an overall impact benefit avoiding food loss of about 23%.

No considerations shall be done for the nutritional parameter, the gap of TPC between the two products was quantified in 413 times (Table 1). Then, the convenience of choosing the enriched mayonnaise is directly quantified in 413 times.

#### 3.1.3. Salad Dressing Impact Assessment

**Figure 7:**
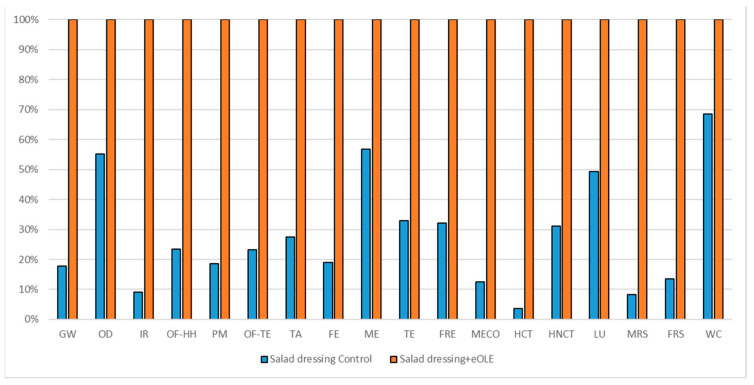
Environmental impact comparison of salad dressing (135 g) vs. PE enriched product. Global warming (GW), Stratospheric ozone depletion (OD), Ionizing radiation (IR), Ozone formation, human health (OF-HH), Fine particulate matter formation (PM), Ozone formation, terrestrial ecosystems (OF-TE), Terrestrial acidification (TA), Freshwater eutrophication (FE), Marine eutrophication (ME), Terrestrial ecotoxicity (TE), Freshwater ecotoxicity (FRE), Marine ecotoxicity (MECO), Human carcinogenic toxicity (HCT), Human non-carcinogenic toxicity (HNCT), Land use (LU), Mineral resource scarcity (MRS), Fossil resource scarcity (FRS), and Water consumption (WC).

The salad dressing shows a simple formulation, which does not require particular ingredients or transformation, highlighting a large benefit in choosing the traditional salad dressing rather than the enriched one if considering the commercial unit (135 g) (Figure 7 and Table S2).

The use of eOLE in the enriched salad dressing formulation represents a high risk for the environment if compared to the traditional salad dressing due to the simpleness of the formulation. The eOLE as an ingredient (as reported in Table 11) shows an overall impact among impact categories equal to 73.51%. The high level of contribution of the eOLE in the enriched product gives the salad dressing + eOLE 72% more impact than the traditional product. Opposite consideration shall be done if the TPC is considered (Table 1): in this case, the traditional formulation does not result in convenient with respect to the enriched one, showing a higher environmental impact of about 96%.

#### 3.1.4. Biscuits Impact Assessment

**Figure 8:**
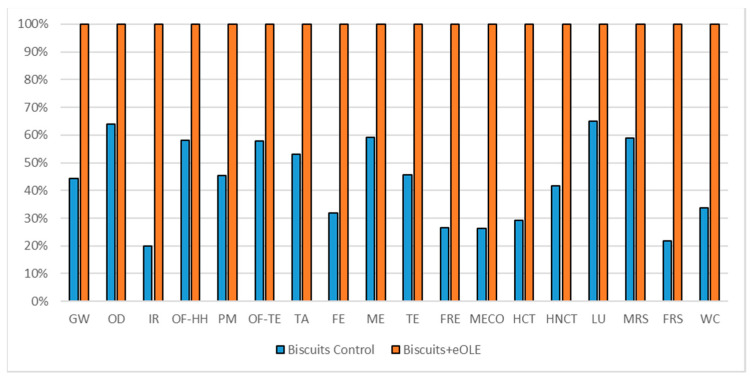
Environmental impact comparison of biscuits (160 g) vs. PE enriched product. Global warming (GW), Stratospheric ozone depletion (OD), Ionizing radiation (IR), Ozone formation, human health (OF-HH), Fine particulate matter formation (PM), Ozone formation, terrestrial ecosystems (OF-TE), Terrestrial acidification (TA), Freshwater eutrophication (FE), Marine eutrophication (ME), Terrestrial ecotoxicity (TE), Freshwater ecotoxicity (FRE), Marine ecotoxicity (MECO), Human carcinogenic toxicity (HCT), Human non-carcinogenic toxicity (HNCT), Land use (LU), Mineral resource scarcity (MRS), Fossil resource scarcity (FRS), and Water consumption (WC).

**Figure 9:**
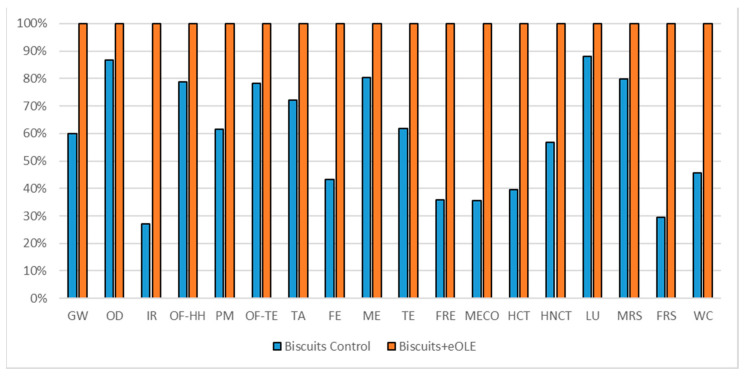
Environmental impact comparison of biscuits vs. PE enriched product normalized to potential shelf-life. Global warming (GW), Stratospheric ozone depletion (OD), Ionizing radiation (IR), Ozone formation, human health (OF-HH), Fine particulate matter formation (PM), Ozone formation, terrestrial ecosystems (OF-TE), Terrestrial acidification (TA), Freshwater eutrophication (FE), Marine eutrophication (ME), Terrestrial ecotoxicity (TE), Freshwater ecotoxicity (FRE), Marine ecotoxicity (MECO), Human carcinogenic toxicity (HCT), Human non-carcinogenic toxicity (HNCT), Land use (LU), Mineral resource scarcity (MRS), Fossil resource scarcity (FRS), and Water consumption (WC).

**Figure 10:**
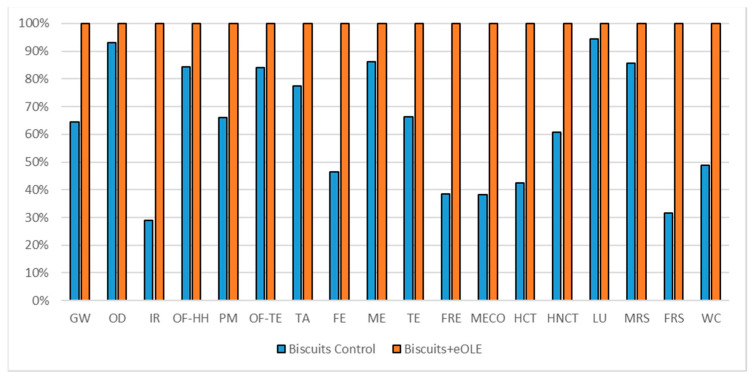
Environmental impact comparison of a vegan mayonnaise vs. PE enriched product normalized to TPC. Global warming (GW), Stratospheric ozone depletion (OD), Ionizing radiation (IR), Ozone formation, human health (OF-HH), Fine particulate matter formation (PM), Ozone formation, terrestrial ecosystems (OF-TE), Terrestrial acidification (TA), Freshwater eutrophication (FE), Marine eutrophication (ME), Terrestrial ecotoxicity (TE), Freshwater ecotoxicity (FRE), Marine ecotoxicity (MECO), Human carcinogenic toxicity (HCT), Human non-carcinogenic toxicity (HNCT), Land use (LU), Mineral resource scarcity (MRS), Fossil resource scarcity (FRS), and Water consumption (WC).

The comparisons of the two biscuits formulations are reported in Figure 8 (considering 160 g commercial unit), in Figure 9 (considering oxidation induction time parameter), and in Figure 10 (considering TPC parameter). All the environmental impacts related to the three normalization parameters are reported together in Table S3.

As for the salad dressing, also for the biscuits, which represent a more complex formulation, the high energy demand coming from the OLE extraction and encapsulation process shows higher environmental impacts for the enriched formulations in all the impact categories. The eOLE ingredient required for the commercial unit is about 0.55 g, which represents a very tiny quantity but reports a high level of contribution as reported in Table 12 (56% among impact categories).

The comparison of the two biscuits formulations (reported in Figure 8) considering a 160 g commercial unit showed that the enriched biscuits formulation impact 56% more with respect to the traditional one (average value among impact categories).

Considering the oxidation induction time parameter (Figure 9), which implies a delta between the two formulations equal to 1.3559, the traditional formulation reports again better environmental impact in all the impact categories (41% less with respect the enriched one). The better behavior of the traditional biscuits is largely highlighted in those impact categories that are directly linked to the energy consumption as IR, HNCT, HCT, and FRS (62% average benefit), while the lowest advantages in choosing the traditional biscuits can be seen in those impact categories linked to the agricultural activities as OD, ME, and LU (15% average benefit).

As reported in Table 6, also considering the TPC normalization parameter, the benefit of the traditional biscuits is confirmed again even if it decreased with respect to the commercial functional unit comparison (37% less). Even if an increase of the relative environmental impact can be registered due to a 1.45 gap (Table 1) between traditional and enriched formulation, the enriched product represents the worst product.

#### 3.1.5. Gluten-Free Breadsticks Impact Assessment

**Figure 11:**
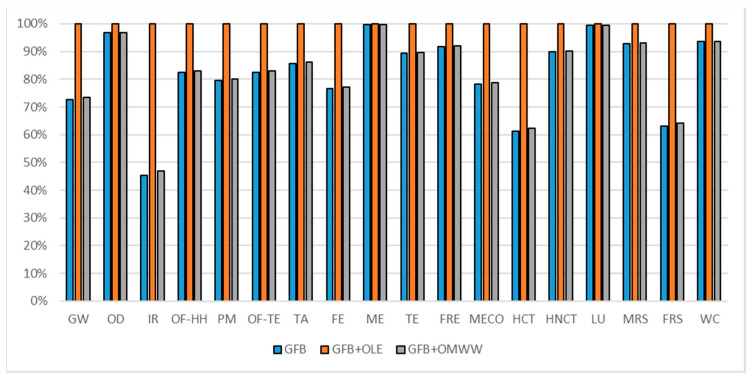
Environmental impact comparison of gluten-free breadsticks (300 g) vs. PE enriched product. Global warming (GW), Stratospheric ozone depletion (OD), Ionizing radiation (IR), Ozone formation, human health (OF-HH), Fine particulate matter formation (PM), Ozone formation, terrestrial ecosystems (OF-TE), Terrestrial acidification (TA), Freshwater eutrophication (FE), Marine eutrophication (ME), Terrestrial ecotoxicity (TE), Freshwater ecotoxicity (FRE), Marine ecotoxicity (MECO), Human carcinogenic toxicity (HCT), Human non-carcinogenic toxicity (HNCT), Land use (LU), Mineral resource scarcity (MRS), Fossil resource scarcity (FRS), and Water consumption (WC).

**Figure 12:**
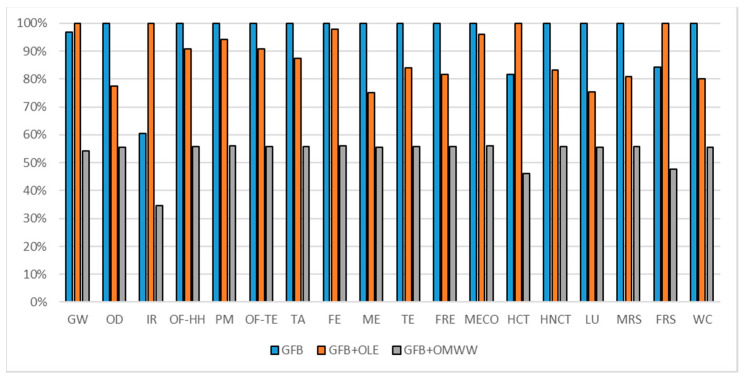
Environmental impact comparison of gluten-free breadsticks vs. PE-enriched product normalized to potential shelf life. Global warming (GW), Stratospheric ozone depletion (OD), Ionizing radiation (IR), Ozone formation, human health (OF-HH), Fine particulate matter formation (PM), Ozone formation, terrestrial ecosystems (OF-TE), Terrestrial acidification (TA), Freshwater eutrophication (FE), Marine eutrophication (ME), Terrestrial ecotoxicity (TE), Freshwater ecotoxicity (FRE), Marine ecotoxicity (MECO), Human carcinogenic toxicity (HCT), Human non-carcinogenic toxicity (HNCT), Land use (LU), Mineral resource scarcity (MRS), Fossil resource scarcity (FRS), and Water consumption (WC).

**Figure 13:**
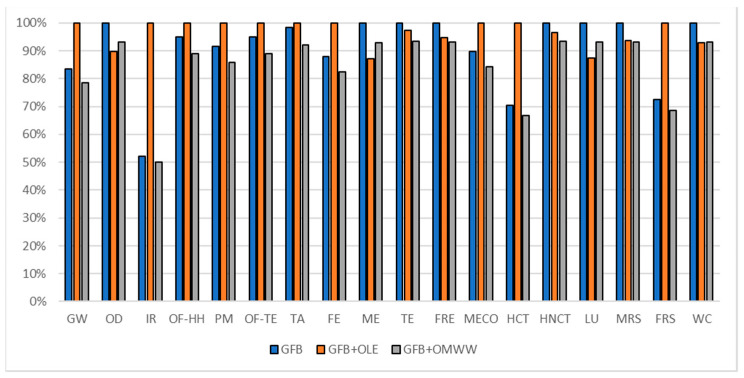
Environmental impact comparison of gluten-free breadsticks vs. PE-enriched product normalized to TPC. Global warming (GW), Stratospheric ozone depletion (OD), Ionizing radiation (IR), Ozone formation, human health (OF-HH), Fine particulate matter formation (PM), Ozone formation, terrestrial ecosystems (OF-TE), Terrestrial acidification (TA), Freshwater eutrophication (FE), Marine eutrophication (ME), Terrestrial ecotoxicity (TE), Freshwater ecotoxicity (FRE), Marine ecotoxicity (MECO), Human carcinogenic toxicity (HCT), Human non-carcinogenic toxicity (HNCT), Land use (LU), Mineral resource scarcity (MRS), Fossil resource scarcity (FRS), and Water consumption (WC).

The gluten-free breadsticks formulation comparisons are reported in Figure 11 (considering a 300 g commercial unit), in Figure 12 (considering oxidation induction time parameter) and in Figure 13 (considering TPC parameter). All the environmental impacts related to the three normalization parameters are reported together in Table S4.

According to the comparison of the commercial unit (300 g of product for each formulation), the OLE-enriched product reports higher environmental impacts in all the impact categories. As reported in Figure 10, the OMWW PE-enriched product reports lower environmental impact with respect to the OLE-enriched one (17% less on average) but higher with respect to the traditional formulation (1% more on average). The average percentage of responsibility related to the OLE in the enriched formulation was a weight of about 17% (as reported in Table 11) while the responsibility related to the OMWW PE in the enriched formulation weight was less than 1% (as reported in Table 11). The gap among the three formulations is highlighted majorly in impact categories directly related to the energy consumption as IR, HNCT, HCT, and FRS where the OLE ingredient has a higher contribution (as reported in Table 11). A consideration regarding only the commercial unit puts in first place the traditional GFB, in second place GFB + OMWWPE, and in third place the OLE enriched one.

Different results are deductible according to the estimated potential shelf life and TPC parameters as reported in Table 7. The overall benefit of potential shelf-life extension enriching the GFB control with OMWW PE can be identified in a potential environmental impact reduction of about 46%. The traditional GFB and the GFB + OLE cannot be defined as the worst solution, both report in some impact categories the higher environmental impact.

Considering the TPC normalization parameter and taking into consideration the average impact among the different impact categories, the differentiation made for the commercial unit cannot be carried out; in some impact categories, the highest level of impact is reached by the GFB control, and in others, the highest level is reached by GFB + OLE. A consideration that should be carried out is that the GFB + OLE on average represents the higher environmental impact formulation, representing the worst choice in most of the impact categories. The choice between the GFB control and GFB + OMWW PE should be linked to the impact category taken into consideration. An overview of the results obtained, considering all three parameters, identifies as the best choice the GFB + OMWW PE, even if in some cases, it means that it is not the best option among the three.

Overall, the results obtained also showed the impact of phenols extracts on the food preparations process considering mean and standard deviation among the impact categories (Table 11). The impact of PE extraction/encapsulation on the whole food chain ranged from 0.71% to 73.51%. This wide range is due to the ingredients and operations provided in the preparation processes. Since the production process consists of a simple formulation in term of (i) ingredients (low quantity and low processed products) and/or (ii) process (few and low energy demand operations), the impact of polyphenols extraction process and encapsulation reached on the whole production process impact a percentage of 73.51%. On the contrary, for those complex preparations, as for the gluten-free breadsticks, the operations relating to phenols extraction process and/or encapsulation weight on the whole process only for 17.72% and 0.71% for GFB + OLE and GFB + OMWW PE respectively, while the impact of phenols extracts on vegan mayonnaise preparation reaches 30.90%.

## 4. Discussion

The results obtained show that the extraction and encapsulation processes, characterized by low production yields, energy-intensive operations, and the partial use of chemical reagents has a non-negligible environmental impact. In detail, to contextualize the results obtained, it is important to analyze the whole supply chain up to the finished product. Even in other critical sectors, such as the plastic packaging field, analyzing only the impact of the material and the production process, the environmental sustainability results are very low [30].

If other aspects were also considered in evaluating the environmental impact, such as the extension of the potential shelf life and therefore the reduction of food waste rather than the entire supply chain [31], the packaging environmental impact would have another weight on the whole supply chain. In this work, after calculating the impact of the polyphenols extraction and encapsulation process from olive oil mill waste, the impact of the extraction process in the food chain was considered, in particular for the production of vegan mayonnaise, biscuits, salad dressing, and gluten-free breadsticks.

The impact of polyphenols extraction/encapsulation on the whole food chain presents very different results based on the operations provided in the formulations process. Considering the advantages in terms of technological and nutritional aspects in the use of enriched formulations, the weight of the polyphenols extraction process and/or encapsulation falls exponentially.

Considering that the LCA evaluation in this work was carried out based on lab-scale data, the impact of the polyphenols extraction process and encapsulation could be reduced in a view of a scale-up process. In fact, the development of pilot plants for the polyphenols extraction and encapsulation within a real chain of reuse of olive oil mill waste would allow the use of more efficient systems and therefore reduce the environmental impact as well as the development of a circular economy model. The environmental impact of the polyphenol extraction and encapsulation process, which for some food preparation showed high contribution, if transferred to a wide context, would allow economic advantages in the valorization of the olive oil supply chain, cancelling out the environmental impact of the polyphenols extraction and encapsulation process from waste.

Nowadays, the olive oil mill by-products are treated with a high energy demand process to transform these products into a second life product (i.e., from pomace to pomace oil, from stone to heat) identifying in the waste product as a high-level product. Other activities are simply catalogued as waste management processes, the output of the oil mill as wastewater, and leaves and dust are treated for composting or fertilizing fields, representing in any case a cost and not a profit for the mill. The revalorization of the waste products coming from the oil milling activities can rearrange all the outputs’ quality level, identifying a profit in waste.

This work should be the basis for future research focused on the environmental impact comparison between the use of phenolic extract deriving from olive oil mill waste and the packaging operations to improve shelf life performance in food chain. The analysis could include different scenarios:

- If the impact on the product of the use of phenolic extract from olive oil milling process wastes results in more sustainability with respect to the use of packaging, it should promote the concept of circular economy. In fact, in this case, there is high probability of a reduction of packaging worldwide re-using olive oil mill waste and adding value to the whole olive oil sector;
- If the use of packaging results is more sustainable than the use of phenolic extracts from olive oil mill wastes, it should be anyway interesting. In this case, the utilization of biocompounds of synthesis instead of phenolic extract from wastes could be the winning choice, reducing the benefits around the food supply chain and having a lower appeal for consumers. It should open further studies to identify the best combo choice between biocompounds and packaging design.

In order to summarize the strengths, weaknesses, opportunities, and threats related to the use of polyphenols extract deriving from olive oil milling waste in the food chain promoting the circular economy, a SWOT table was created (Table 12).

## 5. Conclusions

Nowadays, olive mill waste products could be exploited as by-products to be used as fuels, fertilizers, or other intermediate products for the food, nutraceutical, cosmetic, and pharmaceutical industries. Olive leaf and olive oil mill wastewater have a good potential as sources of bioactive component and could be considered renewable source of high-added value. In this context, the aim of this study is to quantify the environmental impact of the extraction of phenolic compounds from olive oil mill waste. The phenolic extraction and encapsulation obtained from wastewater and olive leaves was characterized by low production yields, energy-intensive operations, and the partial use of chemical reagents. The addition of phenolic extract to food products (vegan mayonnaise, salad dressing, biscuits, and gluten-free breadsticks) leads to enhancing the environmental impact of production process but also implies an improvement of technological and nutritional performance. The potential shelf life of enriched food preparations induces an increase up to two times with respect to control due to the presence of TPC added. This is a crucial aspect to consider in the normalization of environmental impact based on technological and nutritional parameters. This work should be the basis for future research focused on the environmental impact comparison between the use of phenolic extract deriving from olive oil mill waste and the packaging operations to improve shelf life performance in food chain. Moreover, this research can open further studies to identify the best combo choice between biocompounds and packaging design to reduce food waste, reduce packaging, and promote the circular economy business model.

## Figures and Tables

**Table 1:** Technological and nutritional characterization of food preparations.

Products	Oxidative Stability	Polyphenols Content
	Induction days	mg TPC/g product
Mayo control	0.5	0.001
Mayo + OMWW PE	1	0.413
Biscuits control	4.1	1.1
Biscuits + eOLE	5.5	1.6
Salad dressing control	/	0.001
Salad dressing + eOLE	/	0.162
GFB control	84	29.39
GFB + OLE	109	34.08
GFB + OMWW PE	152	31.74

**Table 2:** Economic values and average percentage of mass and economic allocation for the olive oil and by-products of the milling process.

Output	Mass Allocation (%)	Economic Value (€/kg)	Economic Allocation (%)
Olive oil	18.0	3.65	98.33
Pomace	23.0	0.015	0.52
Wastewater (OMWW)	50.0	0.001	0.07
Stone	8.0	0.09	1.08
Leaves (OL) and dust	1.0	0.001	0.00

**Table 3:** Input, output data and allocated quantity per FU, related to the OMWW PE process.

**Input**	**Description**	**Quantity**	**Units**	**Allocation Factor**	**Allocated Quantity per FU**
OMWW	Olive Mill Wastewater	2	L	0.333	0.667
HCl	Acid	1	mL	0.333	0.333
Hexane	2 L × 3 times	6	L	0.004	0.025
Centrifuge	Power load (0.5 kW); use time (9 min)	0.075	kWh	0.333	0.025
Ethyl-acetate	0.625 mL × 3 times	1.875	mL	0.004	0.008
Centrifuge	Power load (0.8 kW); use time (18 min)	0.24	kWh	0.333	0.080
Evaporator	Power load (1 kW); use time (2 h)	2	kWh	0.333	0.667
Water		100	mL	0.333	33.333
Filter		3	g	0.333	1.000
**Waste**	**Description**	**Quantity**	**Units**	**Allocation Factor**	**Allocated Quantity per FU**
Hazardous waste	Hexane	6	L	0.004	0.025
Hazardous waste	Ethyl-acetate	1.875	mL	0.004	0.008

**Table 4:** Input, output data, and allocated quantity per FU, related to the OLE process.

**Input**	**Description**	**Quantity**	**Units**	**Allocation Factor**	**Allocated Quantity per FU**
Olive leaves	Waste from olive oil production	400	g	0.167	66.667
Water	1 L for the washing activity	1	L	0.167	0.167
Oven	Power load (0.530 kW); use time (8 min); Temperature (120 °C); Capacity (400 g)	0.0707	kWh	0.167	0.012
Paper	2–3 pieces	12.5	g	0.167	2.083
Mill	Power load (0.175 kW); use time (30 s)	0.0015	kWh	0.667	0.001
Water	6 L for the extraction	6	L	0.667	4.002
Ultrasound	Power load (0.200 kW); use time (90 min)	0.3000	kWh	0.667	0.200
Filters	1 filter	3	g	0.667	2.001
Freeze dryer	Power load (1.100 kW); use time (8 h)	8.8	kWh	0.667	5.870
**Waste**	**Description**	**Quantity**	**Units**	**Allocation Factor**	**Allocated Quantity per FU**
Biowaste	Exhausted leaves	100	g	0.667	66.700

**Table 5:** Input, output data, and allocated quantity per FU, related to the eOLE process.

**Input Eole**	**Description**	**Quantity**	**Units**	**Allocation Factor**	**Allocated Quantity per FU**
OLE	Olive Leaf Extract	4	g	3.125	12.500
Alginate	Polymer	0.4	g	3.125	1.250
Pectin	Polymer	0.4	g	3.125	1.250
Water		168.92	mL	3.125	527.875
Calcium citrate		1.638	g	3.125	5.119
Sunflower oil	Oil	118	g	3.125	368.750
Span 80	Emulsifier	2	g	3.125	6.250
Stirring plate	Power load (0.4 kW); use time (75 min)	0.5	kWh	3.125	1.563
Glacial acetic acid	Acid	0.5	g	3.125	1.563
Calcium chloride	Gelling agent	0.832	g	3.125	2.600
Tween 20	Surfactant	0.75	g	3.125	2.344
Centrifuge	Power load (0.8 kW); use time (5 min)	0.07	kWh	3.125	0.208
Ethanol		21.00	mL	3.125	65.625
Freeze dryer	Power load (1.100 kW); use time (18 min)	0.33	kWh	3.125	1.031
**Waste**	**Description**	**Quantity**	**Units**	**Allocation Factor**	**Allocated Quantity Per FU**
Hazardous waste	Exhausted oil	300.5	mL	3.125	939.063

**Table 6:** Input, output data, and allocated quantity per FU (350 g), related to the vegan mayonnaise process.

Product	Input	Description	Quantity	Units	Allocation Factor per 350 g Mayo	Allocated Value per 350 g Mayo
Mayo						
control	Soy milk	Ingredient	150	g	1	150
	Sunflower oil	Ingredient	199	g	1	199
	Lemon juice	Ingredient	1	g	1	1
	Blending	Power load (1 kW); use time (4 min)	0.0667	kWh	1	0.0667
Mayo + OMWW PE	Soy milk	Ingredient	100	g	1	100
	OMWW	Ingredient	50	g	1	50
	Sunflower oil	Ingredient	199	g	1	199
	Lemon juice	Ingredient	1	g	1	1
	Blending	Power load (1 kW); use time (4 min)	0.0667	kWh	1	0.0667

**Table 7:** Input, output data, and allocated quantity per FU (135 mL) related to the salad dressing process.

	Input	Description	Quantity	Units	Allocation Factor per 135 mL Salad Dressing	Allocated Quantity per 135 mL Salad Dressing
Salad dressing control	Water	Ingredient	99.55	mL	1	99.55
	Salt	Ingredient	0.40	g	1	0.40
	Xanthan Gum	Ingredient	0.80	g	1	0.80
	Citric acid	Ingredient	0.50	g	1	0.50
	Corn oil	Ingredient	33.75	g	1	33.75
	Blending	Power load (0.75 kW); use time (1 min)	0.0125	kWh	1	0.0125
Salad dressing + eOLE	Water	Ingredient	99.25	mL	1	99.25
	Salt	Ingredient	0.40	g	1	0.40
	Xanthan Gum	Ingredient	0.80	g	1	0.80
	Citric acid	Ingredient	0.50	g	1	0.50
	eOLE	Ingredient	0.30	g	1	0.30
	Corn oil	Ingredient	33.75	g	1	33.75
	Blending	Power load (0.75 kW); use time (1 min)	0.0125	kWh	1	0.0125

**Table 8:** Input, output data, and allocated quantity per FU (160 g), related to the biscuits process.

Product	Input	Description	Quantity	Units	Allocation Factor per 160 g Biscuits	Allocated Quantity per 160 g Biscuits
Biscuits control	Soft wheat flour	Ingredient	100	g	1	100
	Water	Ingredient	17	g	1	17
	Salt	Ingredient	1	g	1	1
	Baking powder	Ingredient	1	g	1	1
	Sugar	Ingredient	50	g	1	50
	Butter	Ingredient	30	g	1	30
	Blending	Power load (0.3 kW); use time (24 min)	0.12	kWh	1	0.12
	Chilling	Energy consumption (250 kWh/year); use time (10 min)	0.0048	kWh	0.01	4.8 × 10^−5^
	Baking	Power load (0.53 kW); use time (20 min)	0.1767			
	kWh	0.25	4.4 × 10^−2^			
Biscuits + eOLE	Soft wheat flour	Ingredient	99.45	g	1	99.45
	eOLE	Ingredient	0.55	g	1	0.55
	Water	Ingredient	17	g	1	17
	Salt	Ingredient	1	g	1	1
	Baking powder	Ingredient	1	g	1	1
	Sugar	Ingredient	50	g	1	50
	Butter	Ingredient	30	g	1	30
	Blending	Power load (0.300 kW); use time (24 min)	0.12	kWh	1	0.12
	Chilling	Energy consumption (250 kWh/year); use time (10 min)	0.005	kWh	0.01	4.8 × 10^−5^
	Baking	Power load (0.530 kW); use time (20 min)	0.177	kWh	0.25	4.4 × 10^−2^
BiscuitscontrolBiscuits + eOLE	Food waste	Production waste	15	g	1	15

**Table 9:** Input, output data, and allocated quantity per FU (300 g), related to gluten-free breadsticks process.

**Product**	**Input**	**Description**	**Quantity**	**Units**	**Allocation Factor per 300 g GFB**	**Allocated Quantity per 300 g GFB**
GFB control; GFB + OLE; GFB + OMWW PE	Rice flour	Ingredient	500	g	0.247	123.457
	Corn starch	Ingredient	500	g	0.247	123.457
	Guar gum	Ingredient	15	g	0.247	3.704
	Psyllium fiber	Ingredient	15	g	0.247	3.704
	Sugar	Ingredient	30	g	0.247	7.407
	Salt	Ingredient	15	g	0.247	3.704
	Compressed yeast	Ingredient	40	g	0.247	9.877
	Sunflower oil	Ingredient	100	g	0.247	24.691
	Water	Ingredient	550	mL	0.247	135.802
	Blending	Power load (0.3 kW); use time (15 min)	0.075	kWh	0.247	0.019
	Leavening chamber	Power load (1.5 kW); (T 33 °C; RH 90%); use time (60 min)	1.5	kWh	0.012	0.019
	Baking	Power load (0.53 kW); use time (35 min)	0.31	kWh	0.062	0.019
GFB + OLE	OLE	OLE	1	g	0.247	0.247
GFB + OMWW PE	OMWW	OMWW	1	g	0.247	0.247
	**Waste**	**Description**	**Quantity**	**Units**	**Allocation Factor per 300 g GFB**	**Allocated Quantity per 300 g GFB**
GFB control; GFB + OLE; GFB + OMWW PE	Food waste	Production waste of raw dough	0.467	g	0.247	0.115

**Table 10:** Environmental impacts of PE extractions and encapsulation processes.

Impact Category	Unit	OMWW PE	OLE	eOLE
GW ^1^	kg CO_2_ eq	4.10 × 10^−1^	2.83	7.84
OD ^2^	kg CFC11 eq	3.32 × 10^−7^	2.34 × 10^−6^	1.63 × 10^−5^
IR ^3^	kBq ^60^Co eq	4.11 × 10^−2^	3.17 × 10^−1^	7.73 × 10^−1^
OF-HH ^4^	kg NO_x_ eq	7.11 × 10^−4^	4.92 × 10^−3^	1.57 × 10^−2^
PM ^5^	kg PM_2.5_ eq	4.52 × 10^−4^	3.29 × 10^−3^	9.97 × 10^−3^
OF-TE ^6^	kg NO_x_ eq	7.40 × 10^−4^	5.00 × 10^−3^	1.60 × 10^−2^
TA ^7^	kg SO_2_ eq	1.38 × 10^−3^	1.01 × 10^−2^	3.29 × 10^−2^
FE ^8^	kg P eq	9.98 × 10^−5^	7.16 × 10^−4^	2.08 × 10^−3^
ME ^9^	kg N eq	9.38 × 10^−6^	5.48 × 10^−5^	4.75 × 10^−3^
TE ^10^	kg 1,4-DCB	4.44 × 10^−1^	2.88	11.1
FRE ^11^	kg 1,4-DCB	1.00 × 10^−2^	6.98 × 10^−2^	3.43 × 10^−1^
MECO ^12^	kg 1,4-DCB	1.30 × 10^−2^	9.05 × 10^−2^	2.79 × 10^−1^
HCT ^13^	kg 1,4-DCB	8.38 × 10^−3^	6.11 × 10^−2^	1.71 × 10^−1^
HNCT ^14^	kg 1,4-DCB	2.02 × 10^−1^	1.43	5.98
LU ^15^	m^2^a crop eq	1.58 × 10^−2^	1.08 × 10^−1^	3.61
MRS ^16^	kg Cu eq	4.16 × 10^−4^	2.56 × 10^−3^	2.00 × 10^−2^
FRS ^17^	kg oil eq	1.29 × 10^−1^	8.37 × 10^−1^	2.25
WC ^18^	m^3^	5.80 × 10^−3^	4.72 × 10^−2^	1.20 × 10^−1^

^1^ Global warming. ^2^ Stratospheric ozone depletion. ^3^ Ionizing radiation. ^4^ Ozone formation, human health. ^5^ Fine particulate matter formation. ^6^ Ozone formation, terrestrial ecosystems. ^7^ Terrestrial acidification. ^8^ Freshwater eutrophication. ^9^ Marine eutrophication. ^10^ Terrestrial ecotoxicity. ^11^ Freshwater ecotoxicity. ^12^ Marine ecotoxicity. ^13^ Human carcinogenic toxicity. ^14^ Human non-carcinogenic toxicity. ^15^ Land use. ^16^ Mineral resource scarcity. ^17^ Fossil resource scarcity. ^18^ Water consumption.

**Table 11:** Mean and standard deviation among the impact categories of extraction and encapsulation processes impact on the whole food chain.

Products	Mean	SD
	%	%
Mayo + OMWW PE	30.90	20.77
Salad dressing + eOLE	73.51	17.22
Biscuits + eOLE	56.61	14.97
GFB + OLE	17.72	14.29
GFB + OMWW PE	0.71	0.75

**Table 12:** Strengths, weaknesses, opportunities, and threats related to use of phenolic extract deriving from the olive oil milling process in food chain.

**Positive**	**Internal**		**Negative**
	Strengths	Weaknesses	
	Reduce waste of olive oil milling process	High environmental impact of extraction/encapsulation processes	
	Transform waste products into a second-life product	Energy-intensive operations to freeze drying operation and encapsulation solvents	
		Chemicals for extraction	
	Opportunities	Threats	
	Promote circular economy business model	Research efforts in optimizing extraction and encapsulation processes in terms of yield extraction	
	Reduce food waste		
	Add values to olive oil sector		
	Design new packaging concept considering the use of bio-compounds to extend shelf-life		
	External		

## Data Availability

Data is contained within the article or supplementary material.

## Supplementary Materials

The following are available online at https://www.mdpi.com/article/10.3390/foods10050980/s1, Figure S1: System boundary, Table S1: Environmental impact comparison of mayonnaise and enriched mayonnaise considering commercial unit, potential shelf-life and TPC parameters, Table S2: Environmental impact comparison of salad dressing and enriched salad dressing considering commercial unit and TPC parameters, Table S3: Environmental impact comparison of biscuits and enriched biscuits considering commercial unit, technological and nutritional parameters, Table S4: Environmental impact comparison of gluten free breadsticks and enriched GFB considering commercial unit, technological and nutritional parameters.
