# Supplementary material for: Environmental Impact of Food Preparations Enriched with Phenolic Extracts from Olive Oil Mill Waste
Source: Foods. 2021 Apr 29;10(5):980. doi: 10.3390/foods10050980 (PMC8147005; doi:10.3390/foods10050980)
Supplement: Supplementary file 1 [file foods-10-00980-s001.zip › Supplementary data/Supplementary data tables.pdf]

**Supplementary materials: Tables**

**Table S1.** Environmental impact comparison of mayonnaise and enriched mayonnaise considering commercial unit, potential shelf-life and TPC parameters.

| | | Commercial unit | | Potential shelf-life | | TPC | |
|---|---|---|---|---|---|---|---|
| Impact category | Unit | Mayo control | Mayo + OMWW PE | Mayo control | Mayo + OMWW PE | Mayo control | Mayo + OMWW PE |
| GW[1] | kg $CO_2$ eq | **6.75 x10$^{-1}$** | 1.26 | 1.35 | **1.26** | 2.79 x10$^{-2}$ | **1.26** |
| OD[2] | kg CFC11 eq | **6.05 x10$^{-6}$** | 6.44 x10$^{-6}$ | 1.21 x10$^{-5}$ | **6.44 x10$^{-6}$** | 2.50 x10$^{-3}$ | **6.44 x10$^{-6}$** |
| IR[3] | kBq $^{60}$Co eq | **2.20 x10$^{-2}$** | 8.16 x10$^{-2}$ | **4.40 x10$^{-2}$** | 8.16 x10$^{-2}$ | 9.09 | **8.16 x10$^{-2}$** |
| OF-HH[4] | kg $NO_x$ eq | **2.27 x10$^{-3}$** | 3.28 x10$^{-3}$ | 4.53 x10$^{-3}$ | **3.28 x10$^{-3}$** | 9.36 x10$^{-1}$ | **3.28 x10$^{-3}$** |
| PM[5] | kg $PM_{2.5}$ eq | **1.26 x10$^{-3}$** | 1.90 x10$^{-3}$ | 2.52 x10$^{-3}$ | **1.90 x10$^{-3}$** | 5.20 x10$^{-1}$ | **1.90 x10$^{-3}$** |
| OF-TE[6] | kg $NO_x$ eq | **2.33 x10$^{-3}$** | 3.38 x10$^{-3}$ | 4.65 x10$^{-3}$ | **3.38 x10$^{-3}$** | 9.61 x10$^{-1}$ | **3.38 x10$^{-3}$** |
| TA[7] | kg $SO_2$ eq | **5.09 x10$^{-3}$** | 7.06 x10$^{-3}$ | 1.02 x10$^{-2}$ | **7.06 x10$^{-3}$** | 2.10 | **7.06 x10$^{-3}$** |
| FE[8] | kg P eq | **2.16 x10$^{-4}$** | 3.59 x10$^{-4}$ | 4.33 x10$^{-4}$ | **3.59 x10$^{-4}$** | 8.94 x10$^{-2}$ | **3.59 x10$^{-4}$** |
| ME[9] | kg N eq | 2.53 x10$^{-3}$ | **2.51 x10$^{-3}$** | 5.06 x10$^{-3}$ | **2.51 x10$^{-3}$** | 1.05 | **2.51 x10$^{-3}$** |
| TE[10] | kg 1,4-DCB | **2.32** | 2.95 | 4.64 | **2.95** | 9.57 x10$^{+2}$ | **2.95** |
| FRE[11] | kg 1,4-DCB | **9.64 x10$^{-2}$** | 1.11 x10$^{-1}$ | 1.93 x10$^{-1}$ | **1.11 x10$^{-1}$** | 3.98 x10$^{+1}$ | **1.11 x10$^{-1}$** |
| MECO[12] | kg 1,4-DCB | **3.59 x10$^{-2}$** | 5.46 x10$^{-2}$ | 7.18 x10$^{-2}$ | **5.46 x10$^{-2}$** | 1.48 x10$^{+1}$ | **5.46 x10$^{-2}$** |
| HCT[13] | kg 1,4-DCB | **1.52 x10$^{-2}$** | 2.72 x10$^{-2}$ | 3.03 x10$^{-2}$ | **2.72 x10$^{-2}$** | 6.26 | **2.72 x10$^{-2}$** |
| HNCT[14] | kg 1,4-DCB | **1.43** | 1.72 | 2.86 | **1.72** | 5.90 x10$^{+2}$ | **1.72** |
| LU[15] | m$^2$a crop eq | **1.78** | **1.78** | 3.55 | **1.78** | 7.34 x10$^{+2}$ | **1.78** |
| MRS[16] | kg Cu eq | **7.49 x10$^{-3}$** | 8.08 x10$^{-3}$ | 1.50 x10$^{-2}$ | **8.08 x10$^{-3}$** | 3.09 | **8.08 x10$^{-3}$** |
| FRS[17] | kg oil eq | **1.39 x10$^{-1}$** | 3.25 x10$^{-1}$ | **2.78 x10$^{-1}$** | 3.25 x10$^{-1}$ | 5.74 x10$^{+1}$ | **3.25 x10$^{-1}$** |
| WC[18] | m$^3$ | **5.65 x10$^{-3}$** | 1.37 x10$^{-2}$ | **1.13 x10$^{-2}$** | 1.37 x10$^{-2}$ | 2.33 | **1.37 x10$^{-2}$** |

[1]Global warming. [2]Stratospheric ozone depletion. [3]Ionizing radiation. [4]Ozone formation, human health. [5]Fine particulate matter formation. [6]Ozone formation, terrestrial ecosystems. [7]Terrestrial acidification. [8]Freshwater eutrophication. [9]Marine eutrophication. [10]Terrestrial ecotoxicity. [11]Freshwater ecotoxicity. [12]Marine ecotoxicity. [13]Human carcinogenic toxicity. [14]Human non-carcinogenic toxicity. [15]Land use. [16]Mineral resource scarcity. [17]Fossil resource scarcity. [18]Water consumption.

**Table S2.** Environmental impact comparison of salad dressing and enriched salad dressing considering commercial unit and TPC parameters.

| | | Commercial unit | | TPC | |
|---|---|---|---|---|---|
| Impact category | Unit | Salad dressing Control | Salad dressing + eOLE | Salad dressing Control | Salad dressing + eOLE |
| GW[1] | kg $CO_2$ eq | **1.01 x10$^{-1}$** | 5.66 x10$^{-1}$ | 1.63 x10$^{+1}$ | **5.66 x10$^{-1}$** |
| OD[2] | kg CFC11 eq | **1.13 x10$^{-6}$** | 2.05 x10$^{-6}$ | 1.83 x10$^{-4}$ | **2.05 x10$^{-6}$** |
| IR[3] | kBq $^{60}$Co eq | **4.66 x10$^{-3}$** | 5.09 x10$^{-2}$ | 7.56 x10$^{-1}$ | **5.09 x10$^{-2}$** |
| OF-HH[4] | kg $NO_x$ eq | **2.84 x10$^{-4}$** | 1.21 x10$^{-3}$ | 4.60 x10$^{-2}$ | **1.21 x10$^{-3}$** |
| PM[5] | kg $PM_{2.5}$ eq | **1.36 x10$^{-4}$** | 7.28 x10$^{-4}$ | 2.20 x10$^{-2}$ | **7.28 x10$^{-4}$** |
| OF-TE[6] | kg $NO_x$ eq | **2.87 x10$^{-4}$** | 1.23 x10$^{-3}$ | 4.65 x10$^{-2}$ | **1.23 x10$^{-3}$** |
| TA[7] | kg $SO_2$ eq | **7.33 x10$^{-4}$** | 2.67 x10$^{-3}$ | 1.19 x10$^{-1}$ | **2.67 x10$^{-3}$** |
| FE[8] | kg P eq | **2.88 x10$^{-5}$** | 1.53 x10$^{-4}$ | 4.67 x10$^{-3}$ | **1.53 x10$^{-4}$** |
| ME[9] | kg N eq | **3.49 x10$^{-4}$** | 6.14 x10$^{-4}$ | 5.65 x10$^{-2}$ | **6.14 x10$^{-4}$** |
| TE[10] | kg 1,4-DCB | **3.20 x10$^{-1}$** | 9.70 x10$^{-1}$ | 5.18 x10$^{+1}$ | **9.70 x10$^{-1}$** |
| FRE[11] | kg 1,4-DCB | **9.45 x10$^{-3}$** | 2.95 x10$^{-2}$ | 1.53 | **2.95 x10$^{-2}$** |
| MECO[12] | kg 1,4-DCB | **2.39 x10$^{-3}$** | 1.90 x10$^{-2}$ | 3.87 x10$^{-1}$ | **1.90 x10$^{-2}$** |
| HCT[13] | kg 1,4-DCB | **3.87 x10$^{-4}$** | 1.06 x10$^{-2}$ | 6.28 x10$^{-2}$ | **1.06 x10$^{-2}$** |
| HNCT[14] | kg 1,4-DCB | **1.58 x10$^{-1}$** | 5.08 x10$^{-1}$ | 2.55 x10$^{+1}$ | **5.08 x10$^{-1}$** |
| LU[15] | $m^2$a crop eq | **2.02 x10$^{-1}$** | 4.10 x10$^{-1}$ | 3.27 x10$^{+1}$ | **4.10 x10$^{-1}$** |
| MRS[16] | kg Cu eq | **1.09 x10$^{-4}$** | 1.30 x10$^{-3}$ | 1.77 x10$^{-2}$ | **1.30 x10$^{-3}$** |
| FRS[17] | kg oil eq | **2.11 x10$^{-2}$** | 1.55 x10$^{-1}$ | 3.42 | **1.55 x10$^{-1}$** |
| WC[18] | $m^3$ | **1.40 x10$^{-2}$** | 2.04 x10$^{-2}$ | 2.26 | **2.04 x10$^{-2}$** |

[1]Global warming. [2]Stratospheric ozone depletion. [3]Ionizing radiation. [4]Ozone formation, human health. [5]Fine particulate matter formation. [6]Ozone formation, terrestrial ecosystems. [7]Terrestrial acidification. [8]Freshwater eutrophication. [9]Marine eutrophication. [10]Terrestrial ecotoxicity. [11]Freshwater ecotoxicity. [12]Marine ecotoxicity. [13]Human carcinogenic toxicity. [14]Human non-carcinogenic toxicity. [15]Land use. [16]Mineral resource scarcity. [17]Fossil resource scarcity. [18]Water consumption.

**Table S3.** Environmental impact comparison of biscuits and enriched biscuits considering commercial unit, technological and nutritional parameters.

| | | Commercial unit | | Potential shelf life | | TPC | |
|---|---|---|---|---|---|---|---|
| Impact category | Unit | Biscuits Control | Biscuits + eOLE | Biscuits Control | Biscuits + eOLE | Biscuits Control | Biscuits + eOLE |
| GW[1] | kg $CO_2$ eq | **$6.76 \times 10^{-1}$** | 1.53 | **$9.17 \times 10^{-1}$** | 1.53 | **$9.83 \times 10^{-1}$** | 1.53 |
| OD[2] | kg CFC11 eq | **$3.14 \times 10^{-6}$** | $4.91 \times 10^{-6}$ | **$4.26 \times 10^{-6}$** | $4.91 \times 10^{-6}$ | **$4.57 \times 10^{-6}$** | $4.91 \times 10^{-6}$ |
| IR[3] | kBq $^{60}$Co eq | **$2.10 \times 10^{-2}$** | $1.05 \times 10^{-1}$ | **$2.84 \times 10^{-2}$** | $1.05 \times 10^{-1}$ | **$3.05 \times 10^{-2}$** | $1.05 \times 10^{-1}$ |
| OF-HH[4] | kg $NO_x$ eq | **$2.36 \times 10^{-3}$** | $4.06 \times 10^{-3}$ | **$3.20 \times 10^{-3}$** | $4.06 \times 10^{-3}$ | **$3.43 \times 10^{-3}$** | $4.06 \times 10^{-3}$ |
| PM[5] | kg $PM_{2.5}$ eq | **$8.99 \times 10^{-4}$** | $1.98 \times 10^{-3}$ | **$1.22 \times 10^{-3}$** | $1.98 \times 10^{-3}$ | **$1.31 \times 10^{-3}$** | $1.98 \times 10^{-3}$ |
| OF-TE[6] | kg $NO_x$ eq | **$2.38 \times 10^{-3}$** | $4.12 \times 10^{-3}$ | **$3.22 \times 10^{-3}$** | $4.12 \times 10^{-3}$ | **$3.46 \times 10^{-3}$** | $4.12 \times 10^{-3}$ |
| TA[7] | kg $SO_2$ eq | **$4.06 \times 10^{-3}$** | $7.63 \times 10^{-3}$ | **$5.50 \times 10^{-3}$** | $7.63 \times 10^{-3}$ | **$5.90 \times 10^{-3}$** | $7.63 \times 10^{-3}$ |
| FE[8] | kg P eq | **$1.06 \times 10^{-4}$** | $3.32 \times 10^{-4}$ | **$1.44 \times 10^{-4}$** | $3.32 \times 10^{-4}$ | **$1.54 \times 10^{-4}$** | $3.32 \times 10^{-4}$ |
| ME[9] | kg N eq | **$7.45 \times 10^{-4}$** | $1.26 \times 10^{-3}$ | **$1.01 \times 10^{-3}$** | $1.26 \times 10^{-3}$ | **$1.08 \times 10^{-3}$** | $1.26 \times 10^{-3}$ |
| TE[10] | kg 1,4-DCB | **1.01** | 2.22 | **1.37** | 2.22 | **1.47** | 2.22 |
| FRE[11] | kg 1,4-DCB | **$1.34 \times 10^{-2}$** | $5.07 \times 10^{-2}$ | **$1.82 \times 10^{-2}$** | $5.07 \times 10^{-2}$ | **$1.96 \times 10^{-2}$** | $5.07 \times 10^{-2}$ |
| MECO[12] | kg 1,4-DCB | **$1.08 \times 10^{-2}$** | $4.11 \times 10^{-2}$ | **$1.46 \times 10^{-2}$** | $4.11 \times 10^{-2}$ | **$1.57 \times 10^{-2}$** | $4.11 \times 10^{-2}$ |
| HCT[13] | kg 1,4-DCB | **$7.60 \times 10^{-3}$** | $2.61 \times 10^{-2}$ | **$1.03 \times 10^{-2}$** | $2.61 \times 10^{-2}$ | **$1.11 \times 10^{-2}$** | $2.61 \times 10^{-2}$ |
| HNCT[14] | kg 1,4-DCB | **$4.66 \times 10^{-1}$** | 1.12 | **$6.32 \times 10^{-1}$** | 1.12 | **$6.78 \times 10^{-1}$** | 1.12 |
| LU[15] | $m^2$a crop eq | **$7.25 \times 10^{-1}$** | 1.12 | **$9.83 \times 10^{-1}$** | 1.12 | **1.05** | 1.12 |
| MRS[16] | kg Cu eq | **$3.10 \times 10^{-3}$** | $5.27 \times 10^{-3}$ | **$4.21 \times 10^{-3}$** | $5.27 \times 10^{-3}$ | **$4.51 \times 10^{-3}$** | $5.27 \times 10^{-3}$ |
| FRS[17] | kg oil eq | **$6.77 \times 10^{-2}$** | $3.12 \times 10^{-1}$ | **$9.17 \times 10^{-2}$** | $3.12 \times 10^{-1}$ | **$9.84 \times 10^{-2}$** | $3.12 \times 10^{-1}$ |
| WC[18] | $m^3$ | **$6.57 \times 10^{-3}$** | $1.95 \times 10^{-2}$ | **$8.91 \times 10^{-3}$** | $1.95 \times 10^{-2}$ | **$9.56 \times 10^{-3}$** | $1.95 \times 10^{-2}$ |

[1]Global warming. [2]Stratospheric ozone depletion. [3]Ionizing radiation. [4]Ozone formation, human health. [5]Fine particulate matter formation. [6]Ozone formation, terrestrial ecosystems. [7]Terrestrial acidification. [8]Freshwater eutrophication. [9]Marine eutrophication. [10]Terrestrial ecotoxicity. [11]Freshwater ecotoxicity. [12]Marine ecotoxicity. [13]Human carcinogenic toxicity. [14]Human non-carcinogenic toxicity. [15]Land use. [16]Mineral resource scarcity. [17]Fossil resource scarcity. [18]Water consumption.

**Table S4.** Environmental impact comparison of gluten free breadsticks and enriched GFB considering commercial unit, technological and nutritional parameters.

| | | Commercial unit | | | Potential shelf life | | | TPC | | |
|---|---|---|---|---|---|---|---|---|---|---|
| Impact category | Unit | GFB control | GFB + OLE | GFB + OMWW PE | GFB control | GFB + OLE | GFB + OMWW PE | GFB control | GFB + OLE | GFB + OMWW PE |
| GW[1] | kg $CO_2$ eq | **2.77 x10$^{-1}$** | 3.82 x10$^{-1}$ | 2.80 x10$^{-1}$ | 4.99 x10$^{-1}$ | 5.15 x10$^{-1}$ | **2.80 x10$^{-1}$** | 3.19 x10$^{-1}$ | 3.82 x10$^{-1}$ | **3.00 x10$^{-1}$** |
| OD[2] | kg CFC11 eq | **2.56 x10$^{-6}$** | 2.64 x10$^{-6}$ | **2.56 x10$^{-6}$** | 4.60 x10$^{-6}$ | 3.57 x10$^{-6}$ | **2.56 x10$^{-6}$** | 2.94 x10$^{-6}$ | **2.64 x10$^{-6}$** | **2.74 x10$^{-6}$** |
| IR[3] | kBq $^{60}$Co eq | **9.76 x10$^{-3}$** | 2.15 x10$^{-2}$ | 1.01 x10$^{-2}$ | 1.76 x10$^{-2}$ | 2.90 x10$^{-2}$ | **1.01 x10$^{-2}$** | 1.12 x10$^{-2}$ | 2.15 x10$^{-2}$ | **1.08 x10$^{-2}$** |
| OF-HH[4] | kg $NO_x$ eq | **8.64 x10$^{-4}$** | 1.05 x10$^{-3}$ | 8.70 x10$^{-4}$ | 1.56 x10$^{-3}$ | 1.41 x10$^{-3}$ | **8.70 x10$^{-4}$** | 9.94 x10$^{-4}$ | 1.05 x10$^{-3}$ | **9.30 x10$^{-4}$** |
| PM[5] | kg $PM_{2.5}$ eq | **4.76 x10$^{-4}$** | 5.98 x10$^{-4}$ | 4.80 x10$^{-4}$ | 8.58 x10$^{-4}$ | 8.07 x10$^{-4}$ | **4.80 x10$^{-4}$** | 5.48 x10$^{-4}$ | 5.98 x10$^{-4}$ | **5.13 x10$^{-4}$** |
| OF-TE[6] | kg $NO_x$ eq | **8.78 x10$^{-4}$** | 1.06 x10$^{-3}$ | 8.83 x10$^{-4}$ | 1.58 x10$^{-3}$ | 1.44 x10$^{-3}$ | **8.83 x10$^{-4}$** | 1.01 x10$^{-3}$ | 1.06 x10$^{-3}$ | **9.45 x10$^{-4}$** |
| TA[7] | kg $SO_2$ eq | **2.24 x10$^{-3}$** | 2.61 x10$^{-3}$ | 2.25 x10$^{-3}$ | 4.02 x10$^{-3}$ | 3.52 x10$^{-3}$ | **2.25 x10$^{-3}$** | 2.57 x10$^{-3}$ | 2.61 x10$^{-3}$ | **2.40 x10$^{-3}$** |
| FE[8] | kg P eq | **8.64 x10$^{-5}$** | 1.13 x10$^{-4}$ | 8.71 x10$^{-5}$ | 1.55 x10$^{-4}$ | 1.52 x10$^{-4}$ | **8.71 x10$^{-5}$** | 9.93 x10$^{-5}$ | 1.13 x10$^{-4}$ | **9.32 x10$^{-5}$** |
| ME[9] | kg N eq | **8.93 x10$^{-4}$** | 8.95 x10$^{-4}$ | **8.93 x10$^{-4}$** | 1.61 x10$^{-3}$ | 1.21 x10$^{-3}$ | **8.93 x10$^{-4}$** | 1.03 x10$^{-3}$ | 8.95 x10$^{-4}$ | **9.56 x10$^{-4}$** |
| TE[10] | kg 1,4-DCB | **8.93 x10$^{-1}$** | 1.00 | 8.96 x10$^{-1}$ | 1.61 | 1.35 | **8.96 x10$^{-1}$** | 1.03 | 1.00 | **9.59 x10$^{-1}$** |
| FRE[11] | kg 1,4-DCB | **2.90 x10$^{-2}$** | 3.16 x10$^{-2}$ | 2.91 x10$^{-2}$ | 5.22 x10$^{-2}$ | 4.27 x10$^{-2}$ | **2.91 x10$^{-2}$** | 3.34 x10$^{-2}$ | 3.16 x10$^{-2}$ | **3.11 x10$^{-2}$** |
| MECO[12] | kg 1,4-DCB | **1.20 x10$^{-2}$** | 1.53 x10$^{-2}$ | 1.21 x10$^{-2}$ | 2.15 x10$^{-2}$ | 2.07 x10$^{-2}$ | **1.21 x10$^{-2}$** | 1.38 x10$^{-2}$ | 1.53 x10$^{-2}$ | **1.29 x10$^{-2}$** |
| HCT[13] | kg 1,4-DCB | **3.58 x10$^{-3}$** | 5.84 x10$^{-3}$ | 3.64 x10$^{-3}$ | 6.45 x10$^{-3}$ | 7.89 x10$^{-3}$ | **3.64 x10$^{-3}$** | 4.12 x10$^{-3}$ | 5.84 x10$^{-3}$ | **3.90 x10$^{-3}$** |
| HNCT[14] | kg 1,4-DCB | **4.74 x10$^{-1}$** | 5.27 x10$^{-1}$ | 4.75 x10$^{-1}$ | 8.53 x10$^{-1}$ | 7.11 x10$^{-1}$ | **4.75 x10$^{-1}$** | 5.45 x10$^{-1}$ | 5.27 x10$^{-1}$ | **5.08 x10$^{-1}$** |
| LU[15] | $m^2$a crop eq | **6.81 x10$^{-1}$** | 6.85 x10$^{-1}$ | **6.81 x10$^{-1}$** | 1.23 | 9.24 x10$^{-1}$ | **6.81 x10$^{-1}$** | 7.83 x10$^{-1}$ | 6.85 x10$^{-1}$ | **7.28 x10$^{-1}$** |
| MRS[16] | kg Cu eq | **1.22 x10$^{-3}$** | 1.31 x10$^{-3}$ | **1.22 x10$^{-3}$** | 2.19 x10$^{-3}$ | 1.77 x10$^{-3}$ | **1.22 x10$^{-3}$** | 1.40 x10$^{-3}$ | **1.31 x10$^{-3}$** | **1.31 x10$^{-3}$** |
| FRS[17] | kg oil eq | **5.31 x10$^{-2}$** | 8.40 x10$^{-2}$ | 5.40 x10$^{-2}$ | 9.55 x10$^{-2}$ | 1.13 x10$^{-1}$ | **5.40 x10$^{-2}$** | 6.10 x10$^{-2}$ | 8.40 x10$^{-2}$ | **5.78 x10$^{-2}$** |
| WC[18] | $m^3$ | **2.55 x10$^{-2}$** | 2.73 x10$^{-2}$ | 2.56 x10$^{-2}$ | 4.59 x10$^{-2}$ | 3.68 x10$^{-2}$ | **2.56 x10$^{-2}$** | 2.94 x10$^{-2}$ | **2.73 x10$^{-2}$** | 2.74 x10$^{-2}$ |

[1]Global warming. [2]Stratospheric ozone depletion. [3]Ionizing radiation. [4]Ozone formation, human health. [5]Fine particulate matter formation. [6]Ozone formation, terrestrial ecosystems. [7]Terrestrial acidification. [8]Freshwater eutrophication. [9]Marine eutrophication. [10]Terrestrial ecotoxicity. [11]Freshwater ecotoxicity. [12]Marine ecotoxicity. [13]Human carcinogenic toxicity. [14]Human non-carcinogenic toxicity. [15]Land use. [16]Mineral resource scarcity. [17]Fossil resource scarcity. [18]Water consumption.
