# Supplementary figures and images for: Environmental Impact of Food Preparations Enriched with Phenolic Extracts from Olive Oil Mill Waste
Source: Foods. 2021 Apr 29;10(5):980. doi: 10.3390/foods10050980 (PMC8147005; doi:10.3390/foods10050980)

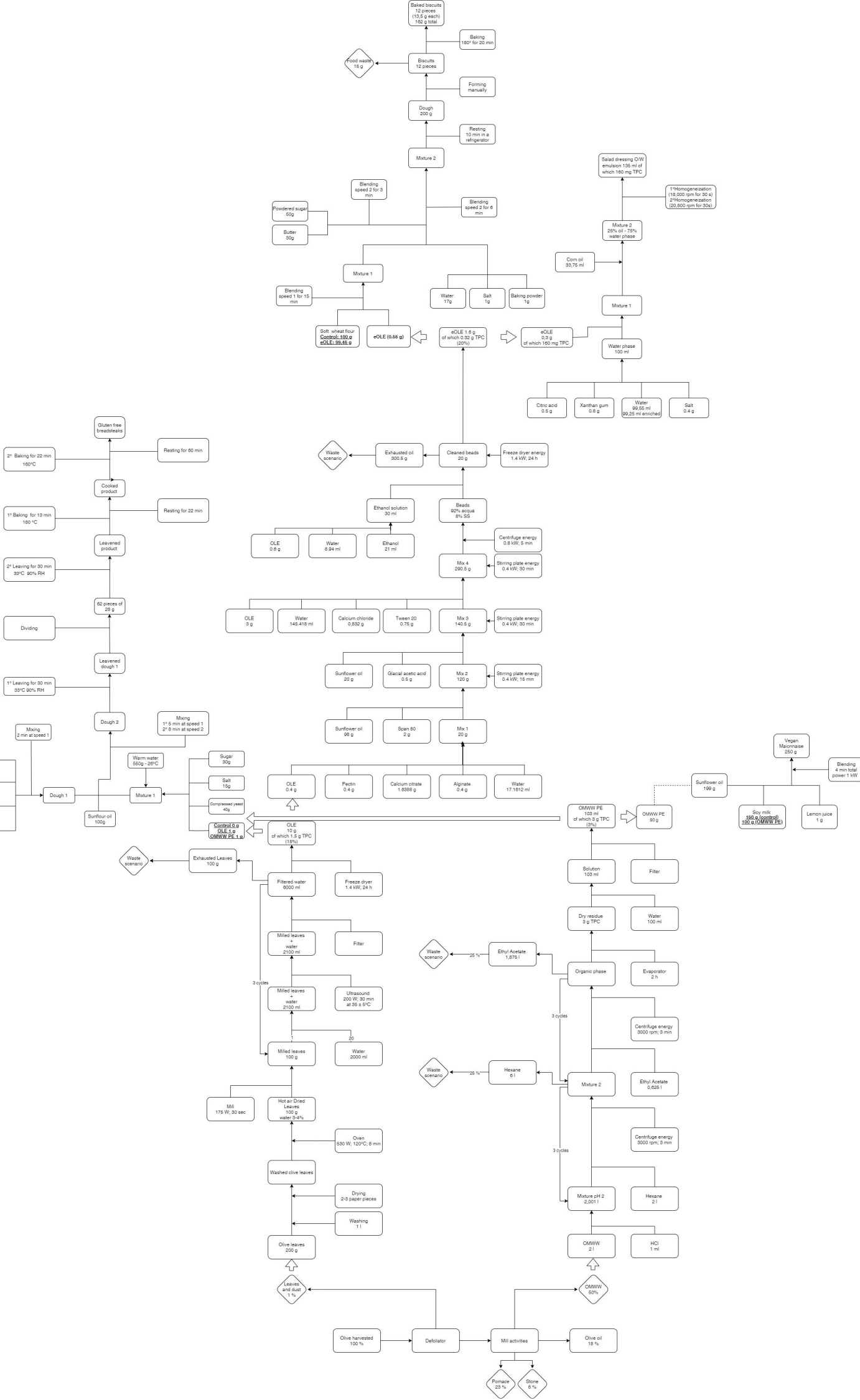

Supplement: Supplementary file 1 [file foods-10-00980-s001.zip › Supplementary data/Supplementary Figure S1 System boundary.pdf]
